# A Comprehensive Survey of Machine Learning Techniques and Models for Object Detection

**DOI:** 10.3390/s25010214

**Published:** 2025-01-02

**Authors:** Maria Trigka, Elias Dritsas

**Affiliations:** Industrial Systems Institute, Athena Research and Innovation Center, 26504 Patras, Greece; trigka@isi.gr

**Keywords:** object detection, machine learning, deep learning, techniques, models

## Abstract

Object detection is a pivotal research domain within computer vision, with applications spanning from autonomous vehicles to medical diagnostics. This comprehensive survey presents an in-depth analysis of the evolution and significant advancements in object detection, emphasizing the critical role of machine learning (ML) and deep learning (DL) techniques. We explore a wide spectrum of methodologies, ranging from traditional approaches to the latest DL models, thoroughly evaluating their performance, strengths, and limitations. Additionally, the survey delves into various metrics for assessing model effectiveness, including precision, recall, and intersection over union (IoU), while addressing ongoing challenges in the field, such as managing occlusions, varying object scales, and improving real-time processing capabilities. Furthermore, we critically examine recent breakthroughs, including advanced architectures like Transformers, and discuss challenges and future research directions aimed at overcoming existing barriers. By synthesizing current advancements, this survey provides valuable insights for enhancing the robustness, accuracy, and efficiency of object detection systems across diverse and challenging applications.

## 1. Introduction

Object detection is a cornerstone of computer vision, focusing on the precise identification and localization of objects within images and videos. This sophisticated technology is fundamental to a wide array of applications across multiple industries. In autonomous vehicles, it is essential for detecting pedestrians, other vehicles, and potential obstacles, ensuring safe navigation. In medical imaging, object detection plays a crucial role in identifying tumours and other abnormalities, significantly aiding in early diagnosis and treatment. Surveillance systems rely on this technology to enhance the monitoring, analysis, and interpretation of activities, providing increased security and situational awareness. Additionally, in industrial settings, object detection is integral to quality control processes, ensuring product standards, and in robotics, where it enables machines to interact more intelligently and accurately with their environment [1].

In recent developments, exponential smoothing has emerged as a powerful image processing technique, particularly when combined with joint power and contrast shrinking in RGB images [2,3]. Moreover, exponential smoothing reduces noise and highlights critical features, leading to enhanced image clarity. This, in turn, allows object detection algorithms to operate more effectively in varied and challenging environments [4]. By refining the visual input, these techniques ensure more precise and reliable detection, which is crucial for maintaining high performance, especially in real-time applications where both accuracy and efficiency are paramount.

The evolution of object detection has been an intriguing journey, beginning with traditional computer vision techniques and advancing through the incorporation of ML and, more recently, DL. Initially, traditional methods were grounded in handcrafted features and simple classifiers, which, despite laying the groundwork for future advancements, struggled to handle the diversity of object appearances and the challenges posed by complex environments. The introduction of ML marked a significant leap forward, bringing with it feature learning methods and more advanced classifiers that enhanced detection capabilities. However, these approaches still encountered limitations when confronted with large-scale datasets and the demands of computational efficiency, necessitating further innovation [5].

DL has radically transformed the field of object detection, offering a powerful means to automatically learn and extract hierarchical features directly from raw data. Central to this revolution are convolutional neural networks (CNNs), which have emerged as the bedrock of contemporary object detection frameworks. CNNs excel in processing and interpreting intricate patterns and structures within images, enabling these systems to achieve unprecedented levels of accuracy and efficiency. This paradigm shift has not only enhanced the precision of object detection but has also dramatically improved processing speed, rendering these systems increasingly suitable for real-time applications across various domains [6].

Object detection encompasses several critical tasks that work in tandem to achieve a detailed and nuanced understanding of visual scenes. The first of these tasks is classification, where the primary objective is to accurately identify and assign a category or label to each detected object within the image. This step is crucial for distinguishing between different types of objects. Next is localization, which focuses on determining the precise location of each object by identifying its coordinates or bounding box within the image. This task ensures that the detected objects are not only recognized but also spatially situated. In some advanced scenarios, segmentation is also employed, which involves delineating the exact boundaries of each object at the pixel level, offering an even more refined understanding of the scene. By integrating these tasks—classification, localization, and segmentation—object detection becomes a comprehensive and sophisticated process, capable of interpreting complex visual environments with high accuracy. This makes it one of the most challenging and vital areas of computer vision [7,8].

The field has witnessed significant advancements through the establishment of influential benchmarks and competitions, including the PASCAL visual object classes (VOC) challenge, the Microsoft COCO (common objects in context) dataset, and the ImageNet large-scale visual recognition challenge (ILSVRC). These initiatives have been pivotal in propelling the progress of object detection, as they offer standardized datasets and rigorous evaluation metrics. By setting high standards and creating a competitive environment, these benchmarks have not only encouraged continuous improvement but also sparked innovation, pushing the boundaries of what is possible in the domain of computer vision [9,10].

Despite the significant advancements in object detection technology, several formidable challenges continue to persist. One of the primary obstacles is managing occlusions, where objects are partially hidden or obscured, making accurate detection difficult. Another challenge lies in addressing varying object scales, where objects appear in different sizes due to changes in perspective or distance, complicating the detection process. The demand for real-time processing adds another layer of complexity, particularly in critical applications such as autonomous driving and live surveillance, where speed and accuracy are paramount. Additionally, achieving consistent and reliable detection in diverse and unstructured environments—where lighting conditions, backgrounds, and object types can vary widely—remains a substantial challenge that the field must overcome to realize its full potential [11,12].

To tackle these challenges head-on, the research community is persistently exploring cutting-edge techniques and models that can revolutionize object detection. Pioneering innovations, including attention mechanisms, Transformer architectures, and sophisticated ML methods, are poised to transcend current limitations, significantly enhancing the accuracy and efficiency of object detection systems. The impetus for conducting this survey is rooted in the fast-paced advancements and the dynamic evolution within the field of object detection. As the landscape continuously shifts with the emergence of novel models and techniques, there is a pressing need for a thorough and cohesive overview that not only synthesizes these developments but also delves into their practical applications and implications.

Several surveys have been conducted on object detection, as summarized in Table 1. These surveys cover a range of topics, such as DL-based object detection methods [13], benchmark datasets, evaluation metrics and lightweight architectures for edge devices [14], metrics for evaluating object detection algorithms [15], the categorization of methods into one-stage and two-stage detectors [16], challenges specific to small object detection, including the introduction of novel datasets [17], and generic object detection techniques focusing on frameworks, proposal generation, and training strategies [18]. However, many of these works focus narrowly on specific aspects of object detection, such as particular methodologies or application domains, without providing a unified perspective on the field’s evolution. Moreover, emerging advancements like Transformer-based architectures and critical challenges, such as occlusion handling and ethical considerations, have not been comprehensively addressed.

**Figure 1:**
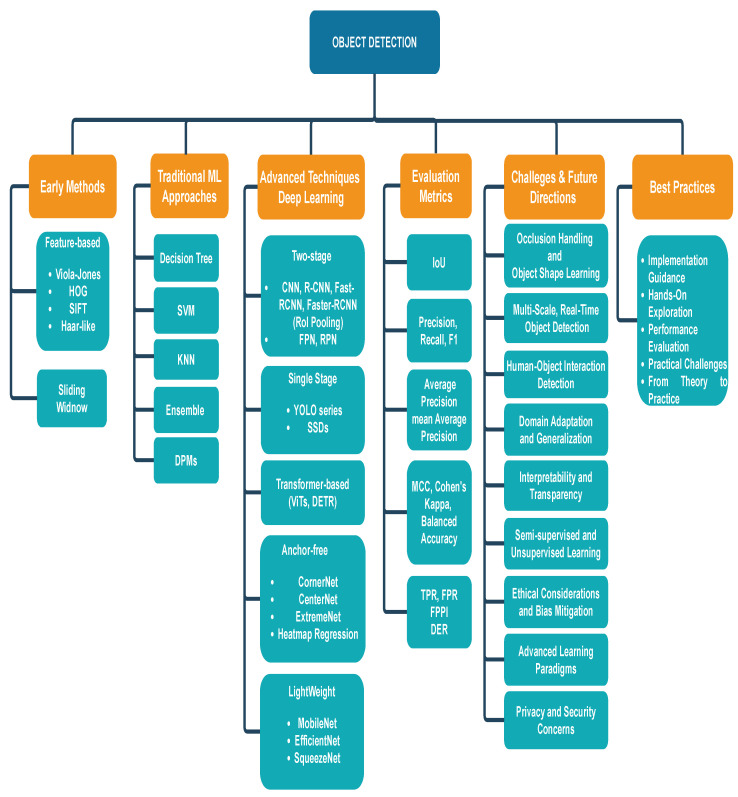
An overview of the object detection landscape.

To bridge the previous gaps, this survey presents a holistic overview of object detection, spanning traditional techniques, modern DL approaches, and future research directions, providing a comprehensive resource for both researchers and practitioners Figure 1 provides a structured roadmap of the object detection domain, showcasing the evolution from initial foundational methods to modern DL architectures. This survey, in particular, aspires to achieve the following objectives:

- Covering the evolution of object detection from traditional methods to modern DL approaches, the survey offers a complete picture of the field’s progression and current state.
- Understanding the strengths and limitations of various techniques and models helps in identifying the most suitable approaches for different applications.
- A detailed examination of evaluation metrics ensures that researchers and practitioners can accurately assess the performance of object detection systems.
- By pinpointing the current challenges and proposing future research directions, the survey aims to inspire new solutions and advancements in the field.

The remainder of the paper is structured as follows. Section 2 provides background knowledge and traditional methods. Section 3 outlines the contribution of DL and its impact on object detection. Section 4 describes techniques and methods for the subject under consideration. In Section 5, the evaluation metrics are noted. Section 6 illustrates challenges and future directions. Section 7 provides a practical guide for implementing and experimenting with the object detection methodologies and techniques discussed in this survey. Finally, Section 8 concludes the present survey.

## 2. Background and Traditional Approaches

The field of object detection has experienced significant evolution, beginning with early techniques that laid the groundwork for future advancements. This section provides an overview of these foundational methods, emphasizing the initially designed approaches before transitioning to more sophisticated ML models. These early techniques, while limited by the technological constraints of their time, were instrumental in addressing the core challenges of object detection, such as feature extraction and classification. The subsequent subsections will explore these pioneering methods, illustrating their development and their role in shaping the modern landscape of object detection technologies.

### 2.1. Early Object Detection Methods

The foundation of object detection in computer vision was built on traditional approaches that relied heavily on handcrafted features and basic classification techniques. In the earliest stages, the primary challenge was how to effectively represent objects in a way that was both computationally feasible and robust to variations in scale, rotation, and illumination.

One of the most influential early methods was the scale-invariant feature transform (SIFT). SIFT was designed to detect and describe local features in images that were invariant to scale and rotation. By identifying key points in an image and generating descriptors that capture local image gradients around those key points, SIFT became a powerful tool for matching objects across different images. Its robustness to changes in viewpoint and illumination made it a popular choice for early object recognition tasks. However, despite its accuracy, SIFT was computationally intensive, which limited its application in real-time systems [19,20,21,22,23].

Around the same time, another critical advancement was the introduction of the histogram of oriented gradients (HOG). HOG features were specifically designed for human detection and became widely used due to their simplicity and effectiveness. The HOG method works by dividing an image into small, connected regions called cells, computing a histogram of gradient directions or edge orientations within each cell, and then normalizing these histograms across blocks of cells. This approach provides a dense grid of feature vectors that capture the underlying structure of objects, making it particularly effective for detecting pedestrians in images. The HOG combination with linear support vector machines (SVMs) in a sliding window approach became the standard pipeline for pedestrian detection. Although HOG was faster than SIFT and more suitable for real-time applications, it still struggled with detecting objects in complex scenes with cluttered backgrounds or under varying lighting conditions [24,25,26,27,28,29].

Another significant development in early object detection was the introduction of Haar-like features. Haar-like features are simple rectangular features used to represent the difference in intensity between adjacent rectangular groups of pixels. These features could be computed rapidly using an integral image, allowing for real-time detection [30,31,32,33,34]. Viola and Jones combined these features with a cascaded classifier, where a series of increasingly complex classifiers were applied sequentially, allowing for efficient object detection, particularly in face detection. The Viola–Jones detector became one of the first successful real-time object detection systems, widely adopted in applications ranging from security cameras to consumer electronics. Despite its success, the Viola–Jones approach was limited by its reliance on rigid, manually designed features, which struggled with variations in pose, scale, and occlusion [35,36,37,38,39].

In parallel to the development of these feature-based methods, the sliding window technique was a common strategy used across various object detection tasks. The sliding window approach involves scanning the entire image at multiple scales and positions and applying a classifier to each window to determine whether an object is present. While conceptually simple, this brute-force method was computationally expensive, as it required evaluating thousands of windows per image, making it impractical for real-time applications. Additionally, the sliding window method was prone to generating a high number of false positives, especially in cluttered environments, which further complicated its application [40,41,42,43,44].

These early methods, outlined in Table 2, laid the groundwork for modern object detection by providing essential insights into feature extraction and classification. However, they also highlighted the limitations of handcrafted features and the need for more sophisticated, automated approaches. The reliance on predefined features meant that these methods often lacked the flexibility to handle the diversity and complexity of real-world data. This recognition would eventually drive the shift toward ML and, later, DL approaches that could learn features directly from the data, leading to significant advancements in detection accuracy and efficiency.

### 2.2. Traditional Machine Learning Approaches

As the limitations of purely feature-based methods became increasingly apparent, the object detection community began to explore more sophisticated techniques that could leverage the power of ML to improve detection accuracy and generalization. The integration of ML into object detection marked a significant turning point, as it allowed models to learn patterns and decision boundaries from data rather than relying solely on handcrafted features. The earliest forays into ML for object detection involved the use of simple classifiers, such as decision trees (DTs), K-nearest neighbors (KNN), and SVMs. These classifiers were often paired with features extracted using methods like SIFT or HOG.

DTs, for instance, provided a straightforward way to model decision boundaries based on feature values. However, while DTs were easy to interpret, they often lacked the robustness required for complex object detection tasks, as they were prone to overfitting, particularly when the training data were limited or noisy [45,46,47,48].

KNN offered another early ML approach, leveraging a non-parametric method that classified objects based on the closest labeled examples in the feature space. While simple and effective for certain tasks, KNN was computationally expensive during inference, as it required comparing each test instance to all training instances. This made KNN less suitable for large-scale or real-time applications, where speed was a critical factor [49,50,51,52].

SVMs emerged as a more powerful alternative, particularly when combined with high-dimensional feature spaces. SVMs aimed to find the optimal hyperplane that maximized the margin between different object classes in the feature space. This approach proved to be highly effective in many object detection tasks, especially when paired with robust feature descriptors like HOG. SVMs could handle non-linear decision boundaries through the use of kernel functions, which allowed them to perform well on complex datasets. However, SVMs required careful tuning of hyperparameters and were computationally intensive, especially when dealing with large datasets or high-dimensional feature spaces [53,54,55,56].

As researchers sought to improve detection accuracy, they turned to ensemble methods, which combined the strengths of multiple classifiers to achieve better performance than any single model. One of the most influential ensemble methods was AdaBoost, a boosting technique that combined several weak classifiers to form a strong classifier. AdaBoost worked by iteratively adjusting the weights of training examples, focusing more on the examples that were misclassified by previous classifiers. This process resulted in a model that was more robust and capable of handling a variety of object detection tasks [57,58,59,60].

The combination of AdaBoost with Haar-like features became particularly famous through the work of Viola and Jones, who developed a highly efficient face detection system. The Viola–Jones detector uses a cascade of classifiers, each trained to detect increasingly complex features, allowing for rapid detection of faces in images. The success of the Viola–Jones detector demonstrated the potential of ML to significantly improve the speed and accuracy of object detection. However, despite its effectiveness for specific tasks like face detection, the Viola–Jones approach was still limited by its reliance on rigid, handcrafted features and struggled with variations in pose, scale, and occlusion [61,62,63,64].

Parallel to these developments, deformable part models (DPMs) introduced a more sophisticated approach by representing objects as collections of parts that could move relative to each other. This part-based model allowed for greater flexibility in detecting objects under different poses and occlusions, making it particularly effective for detecting complex objects like humans and animals. DPMs used latent SVMs to jointly learn the appearance of object parts and their spatial relationships, leading to improved detection accuracy compared to earlier methods. The success of DPMs on benchmarks like PASCAL VOC underscored the potential of combining ML with more structured representations of objects. However, DPMs were still constrained by their reliance on handcrafted features, which limited their ability to scale to more complex and diverse datasets [65,66,67,68]. In Table 3, we summarize recent works that were discussed above and utilize classical ML models for object detection.

The integration of ML into object detection represented a significant advance over previous methods, allowing for more automated and adaptive systems. However, these approaches were not without their limitations. The need for manually designed features and the computational complexity of training and inference posed significant challenges, particularly as datasets grew larger and more diverse. These challenges highlighted the need for further innovation, paving the way for the development of DL methods that could learn both features and classifiers directly from raw data, marking the next major evolution in object detection.

## 3. Deep Learning and Its Impact on Object Detection

DL has revolutionized object detection by enabling models to learn features directly from raw data, vastly improving both accuracy and efficiency. This section explores the evolution of DL in object detection, beginning with the foundational CNNs and their groundbreaking applications, followed by the development of faster, more streamlined frameworks like single shot detectors (SSDs) and You Only Look Once (YOLO), and culminating in the latest advancements with anchor-free detection methods.

### 3.1. CNNs

The introduction of CNNs marked a transformative moment in the field of object detection, fundamentally altering the landscape of computer vision. Unlike traditional methods that depended on manually engineered features, CNNs offered the ability to automatically learn feature hierarchies directly from data. This capability not only improved detection accuracy but also enabled models to generalize better across varying datasets and conditions [69,70,71,72,73].

The breakthrough in using CNNs for object detection began with the development of the regions with convolutional neural networks (R-CNN) model. R-CNN represented a significant departure from earlier methods by leveraging CNNs for feature extraction. The model worked by first generating region proposals from an image, which were then passed through a CNN to extract features. These features were subsequently fed into a classifier to predict the presence and category of objects within each region. This approach demonstrated remarkable improvements in detection accuracy over traditional methods, as CNNs could capture more complex and abstract representations of objects, which were difficult to encode manually [74,75,76,77,78].

However, R-CNN’s performance gains came at the cost of computational efficiency. The need to process each region proposal independently through a CNN led to significant redundancy, as many overlapping regions shared similar features. This inefficiency motivated the development of Fast R-CNN, which introduced several key innovations to streamline the detection pipeline [79,80,81,82]. By employing a technique known as region of interest (RoI) pooling, Fast R-CNN allowed the sharing of convolutional computations across all region proposals. Instead of feeding each proposal through the entire CNN, the model first computed a single feature map for the entire image and then extracted features for each proposal from this shared map. This optimization not only reduced the computational burden but also enabled faster training and inference without sacrificing accuracy [83,84,85,86].

Building upon the successes of R-CNN and Fast R-CNN, the Faster R-CNN model introduced another critical advancement: the region proposal network (RPN). The RPN replaced the external region proposal generation step with a fully integrated, end-to-end trainable network. By generating region proposals directly from the CNN feature map, Faster R-CNN eliminated the need for external proposal algorithms like selective search, further speeding up the detection process. The RPN worked by sliding a small network over the feature map and predicting object bounds and scores at each position. This approach not only streamlined the pipeline but also improved the quality of the proposals, leading to even better detection performance [87,88,89,90]. Faster R-CNN quickly became the benchmark for high-accuracy object detection, setting new standards in various object detection challenges [91,92,93,94,95].

### 3.2. SSDs and YOLO

The pursuit of faster and more efficient object detection models led to the development of SSDs and the YOLO family of models, both of which redefined the landscape of real-time object detection. These models addressed the limitations of earlier region-based methods, such as the R-CNN family, which, despite their high accuracy, were computationally intensive and unsuitable for real-time applications due to their multi-stage pipelines.

SSDs introduced a groundbreaking approach by eliminating the need for separate region proposal stages. SSDs predict both the object classes and bounding boxes directly from feature maps in a single forward pass through the network. This innovation allows for the detection of objects at multiple scales by applying small convolutional filters to different layers of the feature pyramid, each layer responsible for detecting objects of varying sizes. The key advantage of SSDs is their ability to generate a large number of detections across the entire image simultaneously, significantly reducing the computational cost compared to previous methods. By leveraging a fixed set of default boxes with different aspect ratios and scales, SSDs effectively handle the detection of objects at various resolutions within a single network, enabling a more streamlined and efficient detection process [96,97,98,99].

The introduction of YOLO marked another significant advancement in object detection, fundamentally altering how detection tasks were approached. YOLO reframed object detection as a single regression problem, predicting bounding boxes and class probabilities directly from the entire image in one unified process. This end-to-end approach allows YOLO to process images in a single pass, dramatically increasing the speed of detection. The model divides the input image into a grid, with each grid cell predicting a fixed number of bounding boxes and confidence scores for the presence of objects. The simplicity of this approach allows YOLO to operate at an unprecedented speed, achieving real-time performance even on relatively modest hardware. One of the major contributions of YOLO is its unified architecture, which treats object detection as a global problem rather than focusing on individual regions of interest. This holistic view enables YOLO to capture contextual information from the entire image, leading to fewer false positives compared to methods that rely heavily on localized region proposals. However, this design choice also introduced challenges. Early versions of YOLO struggled with small object detection and accurately localizing objects close to each other, primarily due to the coarse grid used for predictions. Additionally, the rigid structure of the grid limited the model’s ability to adapt to objects that did not fit neatly within its predefined cells [100,101,102,103,104].

Despite these challenges, YOLO has seen significant improvements through subsequent iterations. YOLOv2 introduced batch normalization and anchor boxes, which enhanced the model’s ability to detect objects at different scales and improved localization accuracy [105,106,107,108,109]. YOLOv3 [110,111,112,113,114] further refined the architecture by incorporating multi-scale predictions and a deeper, more powerful feature extractor, Darknet-53, allowing the model to balance speed and accuracy more effectively [115,116,117,118].

Moreover, YOLOv4 marked a significant evolution in the YOLO family of object detection models, bringing together a suite of innovations designed to enhance both speed and accuracy [119,120]. The architecture of YOLOv4 integrated cross-stage partial (CSP) connections, which improved the learning process by enabling gradient flow through the network while reducing computation [121]. The backbone of YOLOv4, based on CSPDarknet53, was designed to balance the need for high accuracy and real-time performance [122]. Additionally, YOLOv4 incorporated multiple improvements like the Mish activation function, spatial pyramid pooling (SPP), and path aggregation network (PAN) for better feature fusion, making it one of the most powerful real-time object detectors at its time of release [123,124,125].

YOLOv5, YOLOv6, YOLOv7, and YOLOv8 each built upon the foundation laid by YOLOv4, iterating on architectural advancements to push the boundaries of object detection further. YOLOv5 introduced a more streamlined and user-friendly implementation, focusing on efficiency with a leaner model architecture that maintained competitive performance [126,127]. YOLOv6 continued this trend, emphasizing modularity and scalability, which allowed for tailored deployment in various industrial applications [128,129]. YOLOv7 made strides in optimizing model complexity and inference speed, introducing new techniques like the extended efficient layer aggregation network (E-ELAN) to maximize the use of parameters and computational resources [130,131]. Finally, YOLOv8 integrated these advances into a more sophisticated architecture, refining the network layers and training methods to achieve superior accuracy and faster inference times, positioning it as one of the most advanced models in the YOLO series [132,133,134]. Each iteration of YOLO has pushed the boundaries of what is possible in real-time object detection, making it a popular choice for applications where speed is critical, such as in autonomous driving, robotics, and real-time video analysis.

Both SSD and YOLO represent a shift toward more practical and deployable object detection systems. Their ability to operate at high frame rates without significant sacrifices in accuracy has made these systems highly desirable in environments where computational resources are limited and latency is a critical factor. This has opened up new possibilities for real-time applications, from drone navigation to augmented reality, where traditional, slower object detection methods would be impractical.

### 3.3. Advances in Anchor-Free Detectors

The evolution of object detection has seen a significant shift toward eliminating the reliance on predefined anchor boxes, a core component in many earlier detection frameworks. Anchor boxes, though effective in traditional detectors like Faster R-CNN and SSD, introduced considerable complexity and required meticulous tuning of hyperparameters such as scale, aspect ratio, and the number of anchor boxes per location. Moreover, anchor-based methods often struggled with the inefficiency of matching these predefined boxes to the ground truth, especially when dealing with objects of varying sizes, shapes, and densities. This inherent limitation prompted researchers to explore anchor-free detection methods, which aimed to streamline the detection process and enhance the model’s ability to generalize across different object types and environments [135,136,137,138,139].

The emergence of anchor-free detectors was driven by the need to simplify the detection pipeline while maintaining, or even improving, detection accuracy. These methods bypass the anchor box mechanism entirely by predicting key points, centroids, or other critical features directly from the image, thus reducing the dependence on predefined spatial configurations. One of the pioneering approaches in this category was CornerNet, which re-imagined object detection by framing it as a key point detection problem. Instead of using anchors, CornerNet predicted the top-left and bottom-right corners of bounding boxes and then linked these pairs to form the final detection. This approach offered a more flexible and direct way of localizing objects, particularly excelling in scenarios where objects appeared in non-standard shapes or orientations. By focusing on corners, CornerNet reduced the computational overhead associated with anchor box generation and matching, while also enhancing localization precision [140,141,142,143].

Building upon the concepts introduced by CornerNet, CenterNet further advanced the anchor-free paradigm by simplifying the process to focus on predicting the center point of objects along with their dimensions. CenterNet’s approach was to identify the center of the bounding box and then regress the object’s height and width, effectively capturing the object’s spatial extent without the need for anchors. This method not only simplified the detection process but also improved the model’s efficiency, particularly in detecting objects of various scales within the same image. The elimination of anchors allowed CenterNet to avoid the pitfalls of anchor box design, such as overlapping boxes and the challenge of balancing positive and negative samples during training. This led to more robust and reliable detections across a range of object categories [144,145,146,147,148].

Another notable advance in the anchor-free domain is the concept of dense keypoint estimation, where the model predicts a dense grid of key points across the image, each potentially corresponding to an object’s significant feature, such as its center, edges, or extremities. This approach, exemplified by models like ExtremeNet, extends the idea of corner detection to include additional key points along the boundaries of objects, providing a richer set of cues for object localization. By focusing on key points, these models can effectively handle objects with irregular shapes and complex boundaries, which are often challenging for anchor-based methods [149,150,151,152].

The shift to anchor-free detectors also opened up new possibilities for incorporating advanced techniques like heatmap regression and feature pyramid networks (FPNs), which further enhanced the models’ ability to detect objects at multiple scales and with high precision. Heatmap regression, for instance, allows the model to predict the probability distribution of object key points across the image, effectively capturing the spatial uncertainty and improving localization accuracy. Combined with multi-scale feature extraction techniques, anchor-free models can achieve state-of-the-art performance in scenarios involving small objects, dense object clusters, and varying object sizes [153,154,155,156,157,158,159,160,161].

A synopsis of the above-mentioned classification of DL models for object detection is presented and described in Table 4.

## 4. Advanced Techniques and Methods

As object detection has matured, researchers have sought to push the boundaries of what is achievable by exploring innovative architectures and methodologies that go beyond traditional DL models. These advanced techniques and methods have not only refined detection accuracy and efficiency but also opened new avenues for handling increasingly complex scenarios and challenges in the real world.

One of the most transformative advancements in recent years has been the application of Transformer architectures to object detection [162,163]. Originally developed for natural language processing [164], Transformers have revolutionized tasks such as machine translation and text generation by leveraging self-attention mechanisms to capture long-range dependencies within data [165,166]. Recognizing the potential of these mechanisms, the computer vision community adapted Transformers for image-based tasks, leading to the development of Vision Transformers (ViTs) [167,168] and, more specifically, Detection Transformers (DETR) [169,170]. DETR fundamentally rethinks the object detection pipeline by treating it as a direct set prediction problem, where the model predicts a fixed number of objects without the need for traditional components like anchor boxes or non-maximum suppression. The self-attention mechanism within DETR enables the model to capture complex relationships between objects across the entire image, making it particularly effective in scenarios involving cluttered scenes or overlapping objects. This approach has shown promise in simplifying the object detection pipeline while achieving competitive performance, especially in tasks that require a holistic understanding of the scene [171,172,173,174].

Another significant advancement is the integration of multi-scale and context-aware detection techniques, which address the perennial challenge of detecting objects that vary dramatically in size and appearance. Multi-scale detection, a concept that has evolved over time, has become increasingly sophisticated with the introduction of FPNs. FPNs leverage the inherent hierarchical structure of convolutional neural networks to construct feature maps at multiple levels of abstraction, enabling the detection of small, medium, and large objects with equal proficiency. This approach is particularly advantageous in scenarios such as autonomous driving, where objects of interest can range from distant pedestrians to nearby vehicles. By effectively capturing features across different scales, FPNs enhance the model’s robustness and accuracy, making them a critical component in modern object detection frameworks [175,176,177,178].

The demand for real-time object detection on resource-constrained devices, such as mobile phones and embedded systems, has spurred the development of lightweight models and optimization techniques that maintain high accuracy while minimizing computational requirements [179,180]. Lightweight architectures like MobileNet [181,182], SqueezeNet [183,184], and EfficientNet [185,186] have been specifically designed to operate efficiently on devices with limited processing power and memory. These models achieve remarkable performance by using techniques such as depthwise separable convolutions, which reduce the number of parameters and computations without sacrificing representational power. Additionally, recent advancements in neural architecture search (NAS) have automated the process of discovering optimal network architectures tailored to specific hardware constraints, further enhancing the deployment of object detection models on edge devices [187,188,189].

Optimization techniques such as model pruning, quantization, and knowledge distillation have also become indispensable tools for adapting high-performance models to run on constrained hardware. Model pruning involves systematically removing less critical weights from a neural network, thereby reducing its size and computational complexity [190,191,192]. Quantization reduces the precision of the model’s weights and activations, enabling faster inference with minimal loss of accuracy [193,194]. Knowledge distillation, on the other hand, transfers knowledge from a large, complex model (the teacher) to a smaller, more efficient model (the student), allowing the student model to achieve comparable performance with a fraction of the resources. These techniques collectively enable the deployment of powerful object detection models in real-world applications where computational resources are limited, such as in autonomous drones, IoT devices, and wearable technology [195,196,197,198].

Finally, the exploration of novel modalities and the fusion of multimodal data have opened new frontiers in object detection. While traditional object detection relies primarily on RGB images, integrating additional data types, such as depth maps, infrared imagery, and LiDAR point clouds, can significantly enhance detection performance, particularly in challenging environments. For instance, in low-light conditions or foggy weather, infrared or thermal images can provide critical information that is not visible in the RGB spectrum. Similarly, LiDAR data offer precise distance measurements that can improve the detection and localization of objects in 3D space, which is crucial for applications like autonomous driving and robotics. Multimodal fusion techniques combine these diverse data sources to create a more comprehensive representation of the environment, enabling more robust and accurate object detection across a wide range of scenarios [199,200,201,202,203].

The previously discussed works that analyzed advanced techniques beyond classical DL models (focusing on innovative architectures and methodologies) are presented in Table 5.

## 5. Evaluation Metrics

Evaluation metrics play a pivotal role in the development and assessment of object detection models, providing quantitative measures to compare performance across different methodologies. These metrics provide insight into how well a model detects, localizes, and classifies objects within images, balancing factors such as prediction confidence and error rates. The following illustration delves into the key metrics used in object detection, offering mathematical formulations and theoretical underpinnings to facilitate a deeper understanding of model evaluation [15,204,205,206,207].

Intersection over Union (*IoU*) is a fundamental metric in object detection that measures the overlap between the predicted bounding box and the ground truth bounding box. It is mathematically defined as the ratio of the area of intersection to the area of union between the predicted bounding box ($B_{p}$) and the ground truth bounding box ($B_{gt}$). The formula for IoU is given by the following:

$$IoU=\frac{|B_{p}\cap B_{gt}|}{|B_{p}\cup B_{gt}|},$$

where ∩ denotes the intersection (common area) and ∪ represents the union (total area covered by both boxes). A higher *IoU* indicates a better match between the predicted and actual objects, with values typically ranging from 0 to 1, where 1 represents a perfect match.

Precision quantifies the proportion of true positive predictions among all positive predictions made by the model. It is a measure of the accuracy of positive predictions. Mathematically, precision is defined as the ratio of true positives (TP) to the sum of true positives and false positives (FP), given by the following:

$$\mathrm{Precision}=\frac{TP}{TP+FP}.$$

Precision ranges from 0 to 1, where a precision of 1 indicates that every positive prediction made by the model is correct. Precision is crucial when the cost of false positives is high.

Recall, or sensitivity, measures the proportion of actual positives that are correctly identified by the model. It is a measure of the model’s ability to capture all relevant instances. Recall is mathematically defined as the ratio of true positives (TP) to the sum of true positives and false negatives (FN), expressed as follows:

$$\mathrm{Recall}=\frac{TP}{TP+FN}.$$

Recall ranges from 0 to 1, with a recall of 1 indicating that the model correctly identifies all positive instances. High recall is critical when the cost of false negatives is high, ensuring that the model captures as many true positives as possible.

Average precision (*AP*) is a summary metric that represents the precision across different recall levels, integrating the precision–recall curve. *AP* is calculated as the area under the precision–recall curve, which is computed as the weighted mean of precisions achieved at each threshold, with the change in recall serving as the weight. The formula for *AP* is given by the following:

$$AP=\sum_{n}\left(R_{n}-R_{n-1}\right)P_{n},$$

where $R_{n}$ is the recall at the nth threshold and $P_{n}$ is the precision at the nth threshold. *AP* is a key metric in object detection as it accounts for the trade-off between precision and recall across all thresholds.

Mean average precision (*mAP*) extends the concept of *AP* to multiple object classes, providing a single metric that summarizes the performance across all classes. Mathematically, *mAP* is the mean of the *AP* values calculated for each class, given by the following:

$$mAP=\frac{1}{N}\sum_{i=1}^{N}AP_{i},$$

where *N* is the number of classes and $AP_{i}$ is the average precision for the ith class. *mAP* is widely used in object detection challenges as it provides a comprehensive measure of the model’s ability to detect and localize objects across different categories.

The F1 score is the harmonic mean of precision and recall, providing a single metric that balances both. It is particularly useful when precision and recall are both critical and need to be optimized simultaneously. The F1 score is mathematically defined as follows:

$$F1=2\cdot \frac{\mathrm{Precision}\cdot \mathrm{Recall}}{\mathrm{Precision}+\mathrm{Recall}},$$

which can also be expressed as follows:

$$F1=\frac{2TP}{2TP+FP+FN}.$$

The F1 score ranges from 0 to 1, where a value of 1 indicates perfect precision and recall. It is an important metric when there is an uneven class distribution, as it provides a balanced measure.

The Matthews correlation coefficient (*MCC*) is a metric that provides a more balanced measure of classification quality, taking into account all four quadrants of the confusion matrix (true positives, true negatives, false positives, and false negatives). It is particularly useful for evaluating binary classification tasks with imbalanced classes. *MCC* is mathematically defined as follows:

$$MCC=\frac{TP\times TN-FP\times FN}{\sqrt{\left(TP+FP)(TP+FN)(TN+FP)(TN+FN\right)}}.$$

*MCC* returns a value between −1 and +1, where +1 indicates perfect prediction, 0 signifies no better prediction than random, and −1 indicates complete disagreement between prediction and observation.

Cohen’s Kappa (κ) is a statistical measure of inter-rater agreement that accounts for the possibility of agreement occurring by chance. It is commonly used to assess the performance of models in multi-class classification tasks. The formula for Cohen’s Kappa is given by the following:

$$\kappa =\frac{P_{o}-P_{e}}{1-P_{e}},$$

where $P_{o}$ is the observed agreement (the accuracy of the model) and $P_{e}$ is the expected agreement by chance, calculated as follows:

$$P_{e}=\frac{1}{N^{2}}\sum_{i=1}^{k}\left(A_{i}\cdot B_{i}\right).$$

Here, $A_{i}$ and $B_{i}$ are the marginal totals of the contingency table, *N* is the total number of instances, and *k* is the number of categories. Kappa values range from −1 to 1, where 1 indicates perfect agreement, 0 indicates agreement equivalent to chance, and negative values indicate agreement worse than chance.

Balanced accuracy is a metric designed to address the limitations of traditional accuracy when dealing with imbalanced datasets. It is the average of recall obtained in each class, ensuring that each class contributes equally to the final measure. Balanced accuracy is mathematically defined as follows:

$$\mathrm{Balanced}\;\mathrm{Accuracy}=\frac{1}{2}\left(\frac{TP}{TP+FN}+\frac{TN}{TN+FP}\right).$$

This metric ranges from 0 to 1, where 1 indicates perfect classification across all classes. Balanced accuracy is particularly valuable in scenarios where some classes are underrepresented, providing a more reliable evaluation of model performance.

Localization error measures the discrepancy between the predicted and ground truth bounding boxes. Although no single formula universally defines localization error, it typically involves calculating the Euclidean distance between the centers of the predicted and ground truth boxes, the difference in aspect ratios, and the size differences. This error metric is crucial in object detection tasks where not only the detection but also the precise localization of objects is important.

The confusion matrix is a table that provides a comprehensive summary of the prediction results of a classification problem. It shows the number of correct and incorrect predictions, organized into four categories: true positive (TP), true negative (TN), false positive (FP), and false negative (FN). The confusion matrix is particularly useful for visualizing the performance of a model, especially in multi-class classification problems, by showing the exact count of predicted versus actual instances for each class.

The ROC curve is a graphical representation of the trade-off between the true positive rate (sensitivity) and the false positive rate (1-specificity) across different thresholds. The area under the curve (AUC) quantifies the overall ability of the model to discriminate between the positive and negative classes. The true positive rate (*TPR*) is calculated as follows:

$$TPR=\frac{TP}{TP+FN},$$

and the false positive rate (*FPR*) is calculated as follows:

$$FPR=\frac{FP}{FP+TN}.$$

The AUC is typically computed using numerical integration methods such as the trapezoidal rule, and it ranges from 0 to 1, where 1 indicates a perfect model and 0.5 indicates a model with no discriminative ability.

False positive per image (*FPPI*) measures the average number of false positives detected per image, providing insight into the model’s tendency to make incorrect positive predictions. The formula for FPPI is given by the following:

$$FPPI=\frac{FP}{\mathrm{Number}\;\mathrm{of}\;\mathrm{Images}}.$$

A lower *FPPI* indicates that the model generates fewer false positives, which is desirable in object detection tasks, particularly when the cost of false positives is high.

The objectness score is a metric that represents the likelihood that a region contains an object as opposed to just background. This score is typically output by object detection models like YOLO, which evaluate whether a bounding box contains an object based on the learned features. Although there is no specific formula for the objectness score, it is usually a probability value ranging from 0 to 1, with higher scores indicating a higher likelihood of an object being present.

The detection error rate (*DER*) quantifies the percentage of images in which the model fails to detect all objects or incorrectly detects objects. It is calculated as the sum of false positives and false negatives divided by the total number of detections, expressed as follows:

$$DER=\frac{FP+FN}{\mathrm{Total}\;\mathrm{number}\;\mathrm{of}\;\mathrm{detections}}.$$

*DER* provides a quick summary of the model’s performance, with lower values indicating fewer errors in object detection.

These evaluation metrics provide a comprehensive framework for assessing the performance of object detection models, each offering unique insights into various aspects of model accuracy and reliability. Understanding and utilizing these metrics is crucial for the development of robust and effective object detection systems. This detailed examination of evaluation metrics aims to equip researchers and practitioners with the necessary tools to rigorously assess object detection models, fostering the advancement of the field through precise and meaningful evaluations.

Table 6 organizes the discussed metrics into categories such as classification, localization, detection, and advanced metrics, providing a structured framework to analyze their roles in evaluating object detection models while illustrating the inherent trade-offs between accuracy, computational speed, and error minimization. In particular, Faster R-CNN achieves high mAP and IoU, excelling in classification and localization precision, but its slower inference limits real-time applications. Also, YOLO balances speed and accuracy, achieving competitive precision and recall with lower FPPI, making it suitable for real-time tasks. Moreover, SSD optimizes multi-scale detection, offering strong computational efficiency and accuracy. Error metrics like FPPI and DER provide insights into model reliability, with modern models like Faster R-CNN and DETR showing lower detection errors. Localization metrics, particularly IoU, emphasize the spatial precision achieved by models like Faster R-CNN and DETR. By correlating these metrics with model performance, a comprehensive framework is derived helping to understand the strengths and trade-offs of different detection methods.

## 6. Challenges and Future Directions

Object detection has seen significant advancements over the years, yet it still faces numerous challenges that must be addressed to enable more effective and reliable applications in real-world scenarios.

One of the most persistent challenges in object detection is handling occlusions, where objects are partially obscured by other objects or the environment. Effective occlusion handling requires models to have a robust understanding of context and the ability to infer missing parts of objects. Recent research efforts have focused on learning occluded shapes to enhance 3D object detection, which remains a crucial area for improvement [208,209,210,211].

Another key challenge is detecting objects at multiple scales. In real-world applications, objects often appear in various sizes and perspectives within the same image, making it difficult for models to detect small objects within large scenes and vice versa. Techniques such as multi-scale interactive networks and deep feature learning are being developed to address this issue, offering more sophisticated methods for multi-scale object detection [212,213,214].

The need for real-time processing is particularly critical in applications like autonomous driving, real-time surveillance, and robotics. Balancing accuracy and speed remains a persistent issue, as more complex models tend to slow down processing. Innovations in lightweight networks and optimized inference algorithms are essential to achieve high-performance real-time object detection without sacrificing accuracy [215,216,217].

In the realm of human–object interaction detection, another significant challenge is understanding and predicting interactions between humans and objects. Advances in functional generalization techniques are helping models detect these interactions more accurately, which is essential for applications such as surveillance and human-computer interaction [218,219,220,221].

Object detection in adverse conditions, such as low-light environments or harsh weather, also presents challenges. Thermal object detection using models like YOLO has shown promise in these challenging conditions, enabling better detection performance when traditional methods might fail [222,223,224,225,226]. Domain adaptation and generalization of models across different environments and conditions remain significant hurdles. Models trained on specific datasets often fail to perform well in diverse settings, such as varying lighting conditions, weather, and camera angles. Progressive domain adaptation techniques are being explored to enhance model robustness and adaptability in different environments [227,228,229].

Another pressing issue is the interpretability and transparency of DL models in object detection. As these models are often treated as black boxes, understanding their decision-making processes is challenging. The emerging field of Explainable AI (XAI) introduces methods that enhance the interpretability of object detection models by visualizing and explaining learned features, thereby improving their transparency. This increased transparency aids in diagnosing errors, fostering trust in model predictions, and enhancing overall robustness [230,231,232,233].

The process of data annotation for training high-quality object detection models is both labor-intensive and time-consuming, which poses a bottleneck in model development. To mitigate this, semi-supervised and unsupervised learning methods are being explored. These approaches leverage large amounts of unlabeled data, reducing dependency on extensive labeled datasets while maintaining high performance [234,235,236,237].

Ethical considerations and bias in object detection systems are critical issues that need to be addressed. Bias in training data can lead to models that perform poorly on certain demographic groups or object types, raising concerns about fairness and ethical implications. Ensuring unbiased performance in object detection models requires careful dataset curation and the development of algorithms to detect and mitigate biases [238,239,240].

Looking forward, there are exciting opportunities for integrating advanced learning paradigms such as reinforcement learning, meta-learning, and multi-task learning into object detection. These techniques can help models learn more effectively from their interactions and adapt to new tasks with minimal additional training, pushing the boundaries of what is possible in object detection [241,242,243].

As object detection technology continues to evolve, privacy and security concerns are becoming increasingly important, especially in surveillance and autonomous systems. Addressing these concerns involves developing models that respect privacy while maintaining security, ensuring that the technology is used responsibly [244,245,246,247]. The list of discussed challenges in key topics related to object detection is summarized in Table 7.

Finally, the future of object detection will likely see a convergence of multiple disciplines and technologies. Research will increasingly focus on creating holistic vision systems that integrate object detection with other computer vision tasks, such as instance segmentation and object tracking. This integration could lead to more versatile and powerful systems capable of operating in complex and dynamic environments. Moreover, fostering collaboration between academia, industry, and interdisciplinary fields will be crucial in driving innovation and establishing standardized benchmarks for the future of object detection [248,249,250,251,252].

## 7. Best Practices for Object Detection

This section provides a practical guide for implementing and experimenting with the object detection methodologies and techniques discussed in this paper. While Section 6 outlines the challenges and future directions in the field, the following best practices aim to help researchers and practitioners navigate these obstacles effectively. By applying these recommendations, readers can better understand existing approaches, evaluate their performance, and address common challenges, such as occlusions, multi-scale detection, real-time constraints, and data annotation.

The proposed practices are derived from the methods presented earlier, ranging from traditional approaches to advanced DL models. By systematically engaging with these techniques, researchers can gain hands-on experience, identify the most suitable methods for specific tasks, and contribute to the advancement of robust and efficient object detection systems. These practices provide a structured approach for navigating the complexities of object detection and fostering a deeper understanding of the methodologies surveyed in this paper.

A strong foundation begins with understanding the evolution of object detection, from traditional methods such as Viola–Jones, HOG, and SIFT to modern ML approaches. Revisiting these earlier techniques helps contextualize the strengths and limitations that led to the adoption of DL methods. Researchers are encouraged to begin experimentation with pre-trained models like Faster R-CNN, SSD, and the YOLO series, which represent significant milestones in object detection. These models can be evaluated on benchmark datasets, such as PASCAL VOC and COCO, to gain insights into their performance across different scenarios. Performance evaluation is critical to understanding detection accuracy and localization quality. Researchers should rely on established metrics, such as IoU, AP, and mAP, which have been thoroughly presented in the paper. Using these metrics ensures consistency when comparing models and evaluating improvements.

For addressing the challenges of multi-scale detection, methods like FPN, which have been discussed extensively, provide solutions for detecting objects of varying sizes. Data augmentation strategies—such as scaling, cropping, and flipping—further enhance the robustness of models when dealing with images containing objects of different scales. Handling occlusions, as highlighted among the key challenges, can be approached by exploring advanced techniques such as anchor-free methods like CenterNet, which predict object key points and offer greater flexibility in challenging scenes. Combining these techniques with training strategies that simulate occlusions in the dataset enhances the ability of models to detect partially visible objects effectively.

For real-time applications, lightweight architectures such as MobileNet and EfficientNet offer a balance between speed and accuracy. These methods, coupled with optimization techniques like model pruning and quantization, enable the deployment of high-performing models in resource-constrained environments—essential for applications such as mobile devices or edge computing. Moreover, to overcome the labor-intensive nature of data annotation, leveraging semi-supervised learning methods can reduce dependency on large labeled datasets, making object detection more feasible for researchers with limited resources. Finally, staying current with recent advancements, including the emergence of Transformer-based methods like DETR, aligns with the future directions discussed previously. These methods demonstrate promising potential for addressing challenges such as complex object relationships and cluttered scenes. To ensure transparency and reproducibility, researchers should document their findings and share code using platforms like GitHub or similar repositories.

By following these best practices, researchers can systematically engage with object detection methods, evaluate their performance, and address practical challenges. This structured approach provides a pathway for advancing knowledge and contributing to the ongoing evolution of object detection systems.

## 8. Conclusions

The field of object detection has experienced significant advancements due to the integration of ML and DL techniques. These advancements have led to substantial improvements in accuracy, speed, and robustness, transforming applications ranging from autonomous vehicles to medical imaging. Traditional approaches, which depended on handcrafted features, have been outpaced by techniques that leverage the power of learning algorithms to extract meaningful features and detect objects in complex environments.

DL, particularly through neural network architectures, has driven much of the recent progress. These models have shown remarkable capability in learning hierarchical features directly from data, making them highly effective for diverse and challenging object detection tasks. The continuous innovation in this area includes exploring new architectures and learning paradigms that push the boundaries of performance and efficiency.

Despite the progress, several challenges persist, such as dealing with occlusions, varying object scales, and the demand for real-time processing. Addressing these challenges requires ongoing research into more sophisticated feature extraction methods, improved model interpretability, and the adoption of unsupervised and semi-supervised learning techniques.

In conclusion, the advancements in object detection through ML and DL have been transformative, yet there is still room for improvement. Future research will likely focus on overcoming existing challenges and further refining these technologies to enhance their applicability and effectiveness across various domains. This survey underscores the rapid evolution of the field and provides insights into potential future directions for continued innovation and improvement in object detection systems.

## Figures and Tables

**Table 1:** Overview of surveys covering related topics in object detection.

Reference	Description
[13]	Comprehensive survey of DL-based object detection methods, covering components, strategies, and applications. Highlights advancements in architectures, sampling techniques, and future research directions.
[14]	Discusses benchmark datasets, evaluation metrics, and lightweight networks for object detection, emphasizing performance on edge devices and comparing major architectures.
[15]	Explores metrics and their variants for evaluating object detection algorithms and proposes a standardized implementation for cross-dataset compatibility.
[16]	Reviews state-of-the-art object detection methods, categorizing them into one-stage and two-stage detectors, and discusses their applications in real-world scenarios.
[17]	Focuses on challenges in small object detection, introduces two novel datasets, and evaluates mainstream methods to facilitate progress in this sub-field.
[18]	Covers generic object detection techniques using DL, focusing on frameworks, proposal generation, context modelling, and training strategies. Identifies research trends and challenges.

**Table 2:** Overview of works with early methods in object detection.

Method	References	Description
SIFT	[19,20,21,22,23]	Discuss the development and applications of SIFT, a method for detecting and describing local features in images invariant to scale and rotation.
HOG	[24,25,26,27,28,29]	Focus on HOG, a feature descriptor used for object detection by computing gradients in localized portions of an image, particularly effective for human detection.
Haar-like Features	[30,31,32,33,34]	Explores the use of Haar-like features, which are simple rectangular features used in object detection by comparing pixel intensities, notably applied in face detection.
Viola–Jones detector	[35,36,37,38,39]	Details the Viola–Jones method, which combines Haar-like features with a cascaded classifier for real-time face detection and other object detection tasks.
Sliding Window Method	[40,41,42,43,44]	Describe the Sliding Window technique, a brute-force method for object detection by scanning an image at multiple scales and positions, typically using classifiers.

**Table 3:** Summary of works with traditional ML approaches.

Model	References	Purpose of Use
DT	[45,46,47,48]	DTs are used to model decision boundaries based on feature values. They are easy to interpret but can overfit, especially with limited or noisy data.
KNN	[49,50,51,52]	KNN is a non-parametric method that classifies objects based on the closest labeled examples in the feature space. It is effective but computationally expensive for large datasets.
SVM	[53,54,55,56]	SVMs are powerful for object detection, especially when combined with high-dimensional feature spaces and kernel functions. They require careful tuning and are computationally intensive.
Ensemble Methods	[57,58,59,60,61,62,63,64]	They discuss methods such as voting with NN detectors, ensembling based on class hierarchy, weighted ensemble block and non-maximum suppression ensembling which are used to combine the strengths of multiple classifiers for better performance in object detection. AdaBoost, which combines weak classifiers to form a strong classifier, is applied in various domains: face detection using the Viola–Jones detector, vehicle recognition, and iris localization based on Haar-like features and underwater object detection combined with DL.
DPMs	[65,66,67,68]	DPMs represent objects as collections of parts, allowing for flexibility in detecting objects under various poses and occlusions. They improve detection accuracy in complex scenarios.

**Table 4:** Summary of works related to DL and object detection.

Model	References	Description
CNN	[69,70,71,72,73]	Discuss the foundational role of CNNs in object detection, focusing on models, methodologies, and various applications.
R-CNN	[74,75,76,77,78]	Cover the evolution of R-CNN models including original R-CNN, Fast R-CNN, and related advancements in region-based detection.
Fast R-CNN	[79,80,81,82]	Examine Fast R-CNN, focusing on improvements in efficiency and accuracy by sharing computations across regions.
Faster R-CNN	[91,92,93,94,95]	Discuss Faster R-CNN, which uses RPN for faster and more accurate object detection.
RoI Pooling	[83,84,85,86]	Detail the use of RoI pooling in enhancing object detection efficiency and performance in various models.
RPN	[87,88,89,90]	Focuses on advancements in RPN technology, contributing to faster and more integrated object detection pipelines.
SSD	[96,97,98,99]	Discuss SSDs, which eliminate region proposal stages for faster object detection across multiple scales.
YOLO Family	[100,101,102,103,104,105,106,107,108,109,110,111,112,113,114,119,120,121,122,123,124,125,126,127,128,129,130,131,132,133,134]	Cover the YOLO series, from the original YOLO to YOLOv8, detailing the innovations that allow for real-time object detection.
Darknet-53	[115,116,117,118]	Describe the Darknet-53 architecture, highlighting its balance of speed and accuracy.
Anchor-Free Detectors	[135,136,137,138,139]	Discuss the shift toward anchor-free object detection methods, focusing on reducing complexity and improving accuracy.
CornerNet	[140,141,142,143]	Focuses on CornerNet, an anchor-free detector that uses keypoint detection to identify object corners.
CenterNet	[144,145,146,147,148]	Examines CenterNet, which simplifies object detection by focusing on predicting the center of objects.
ExtremeNet	[149,150,151,152]	Describes ExtremeNet, a model that extends keypoint detection to include object extremities for better localization.
Heatmap regression	[153,154,155,156]	Discusses heatmap regression techniques used for predicting key points in anchor-free models.
FPN	[157,158,159,160,161]	Covers advancements in feature pyramid networks, which enhance multi-scale object detection, particularly for small objects.

**Table 5:** A list of advanced techniques and methods beyond classical DL models.

Technique	References	Description
Transformer Architectures	[162,163,164,165,166,167,168,169,170,171,172,173,174]	Focus on the application of Transformer models to object detection, such as ViTs and DETR, revolutionizing detection by leveraging self-attention mechanisms.
Multi-Scale and Context-Aware Detection	[175,176,177,178]	Discuss the integration of multi-scale detection techniques like FPNs to handle objects of varying sizes and appearances efficiently.
Lightweight Models and Optimization	[179,180,181,182,183,184,185,186,187,188,189]	Cover the development of lightweight architectures like MobileNet, SqueezeNet, EfficientNet, and optimization techniques such as pruning, quantization, and NAS for real-time object detection on resource-constrained devices.
Model Pruning and Quantization	[190,191,192,193,194]	Detail methods for simplifying models through pruning and quantization to enable deployment on devices with limited computational resources.
Knowledge Distillation	[195,196,197,198]	Explore techniques for transferring knowledge from larger models to smaller ones to maintain performance while reducing computational demands.
Multimodal Fusion	[199,200,201,202,203]	Discuss the fusion of data from different modalities, such as LiDAR, infrared, and RGB, to improve detection in challenging environments.

**Table 6:** Grouped evaluation metrics in object detection.

Category	Metrics
Classification	Precision
	Recall (Sensitivity)
	F1-Score
Localization	IoU
Detection	mAP, DER, FPPI
Agreement	κ, MCC
Advanced	Balanced Accuracy
	ROC-AUC

**Table 7:** A taxonomy of challenges, key topics, and reference works.

Challenge	References	Key Topics/Descriptions
Occlusion Handling and Object Shape Learning	[208,209,210,211]	Learning occluded shapes for 3D object detection.
Multi-scale object detection	[212,213,214]	Multi-scale interactive networks and deep feature learning for object detection.
Real-time object detection	[215,216,217]	Lightweight networks and optimized inference algorithms for real-time object detection.
Human–object interaction detection	[218,219,220,221]	Detecting human–object interactions via functional generalization.
Thermal Object Detection	[222,223,224,225,226]	Using YOLO for object detection in challenging weather conditions.
Domain adaptation and Generalization	[227,228,229]	Progressive domain adaptation for object detection in various environments.
Interpretability and transparency	[230,231,232,233]	Interpretable DL models and methods for visualizing and interpreting features.
Semi-supervised and Unsupervised Learning	[234,235,236,237]	Leveraging unlabeled data to reduce dependency on labeled datasets for object detection.
Ethical considerations and Bias mitigation	[238,239,240]	Addressing bias in object detection systems and ensuring fairness across different groups.
Advanced learning paradigms	[241,242,243]	Integration of reinforcement learning, meta-learning, and multi-task learning in object detection.
Privacy and security concerns	[244,245,246,247]	Addressing privacy and security concerns in object detection applications.
